# Plastid phylogenomics contributes to the taxonomic revision of taxa within the genus *Sanicula* L. and acceptance of two new members of the genus

**DOI:** 10.3389/fpls.2024.1351023

**Published:** 2024-06-10

**Authors:** Bo-Ni Song, Chang-Kun Liu, Ting Ren, Yu-Lin Xiao, Lian Chen, Deng-Feng Xie, An-Guo He, Ping Xu, Xing Fan, Song-Dong Zhou, Xing-Jin He

**Affiliations:** ^1^ Key Laboratory of Bio-Resources and Eco-Environment of Ministry of Education, College of Life Sciences, Sichuan University, Chengdu, China; ^2^ CAS Key Laboratory of Mountain Ecological Restoration and Bioresource Utilization & Ecological Restoration and Biodiversity Conservation Key Laboratory of Sichuan Province, Chengdu Institute of Biology, Chinese Academy of Sciences, Chengdu, China; ^3^ College of Resources Environment and Chemistry, Chuxiong Normal University, Chuxiong, China; ^4^ Administration of Zhejiang Dapanshan National Nature Reserve, Zhejiang, China; ^5^ Chengdu Branch of Giant Panda National Park, Chengdu, China

**Keywords:** *Sanicula*, phylogenomics, plastome, variety, new species, taxonomic revision

## Abstract

**Introduction:**

The genus *Sanicula* L. is a taxonomically complicated taxa within Apiaceae, as its high variability in morphology. Although taxonomists have performed several taxonomic revisions for this genus, the interspecific relationships and species boundaries have not been satisfactorily resolved, especially for those endemic to China. This study mainly focused on *S*. *giraldii* var. *ovicalycina*, *S*. *tienmuensis* var. *pauciflora*, and *S. orthacantha* var. *stolonifera* and also described two new members of the genus.

**Methods:**

We newly sequenced sixteen plastomes from nine *Sanicula* species. Combined with eleven plastomes previously reported by us and one plastome downloaded, we performed a comprehensively plastid phylogenomics analysis of 21 *Sanicula* taxa.

**Results and Discussion:**

The comparative results showed that 21 *Sanicula* plastomes in their structure and features were highly conserved and further justified that two new species were indeed members of *Sanicula.* Nevertheless, eleven mutation hotspot regions were still identified. Phylogenetic analyses based on plastome data and the ITS sequences strongly supported that these three varieties were clearly distant from three type varieties. The results implied that these three varieties should be considered as three independent species, which were further justified by their multiple morphological characters. Therefore, revising these three varieties into three independent species was reasonable and convincing. Moreover, we also identified and described two new *Sanicula* species (*S. hanyuanensis* and *S. langaoensis*) from Sichuan and Shanxi, China, respectively. Based on their distinct morphological characteristics and molecular phylogenetic analysis, two new species were included in *Sanicula*. In summary, our study impelled the revisions of *Sanicula* members and improved the taxonomic system of the genus.

## 1 Introduction

*Sanicula* L. is a distinctive genus of Apiaceae subfamily Saniculoideae with high medicinal value (Pryer and Phillippe, 1989). The genus comprises approximately 45 species that are widely distributed from East Asia to North America, with China and North America as two diversification centers (Van et al., 2013; Li et al., 2023). Among them, nineteen species and five varieties are distributed in China and eleven species and five varieties are endemic (Sheh and Phillippe, 2005; Pimenov, 2017; Xie et al., 2019; Li et al., 2023; Song et al., 2024). The most distinctive characteristic features of the genus are the fruits (mericarps) covered with scales, bristles, or hooked prickles, a rather prominent and persistent calyx, and two persistent styles that can easily distinguish it from other genera of Apiaceae (Pryer and Phillippe, 1989; Calviño and Downie, 2007). Published studies illustrated that *Sanicula* was closely related to the genus *Eryngium* L. However, *Eryngium* has its distinctive morphological features, such as capitate inflorescences and single bract per flower, which is easily distinguished from *Sanicula* (Vargas et al., 1998; Valiejo-Roman et al., 2002; Calviño and Downie, 2007; Calviño et al., 2008). Traditionally, plant taxonomists tended to study the genus based on morphological characteristics and to divide the genus into more smaller classification units, whereas many members of the genus always exhibited varied morphological features in rhizomes, foliage, flowers, and fruits (Shan and Constance, 1951), which have resulted in massive disagreements over classification system (De Candolle, 1830; Drude, 1898; Wolff, 1913; Shan and Constance, 1951). In addition, species relationships and species identification in the genus were also blurred, largely due to phenotypic plasticity or the lack of taxonomically robust morphological characters at the species level (Pryer and Phillippe, 1989; Vargas et al., 1999; Calviño and Downie, 2007; Pimenov, 2017; Li and Song, 2022; Li et al., 2022). For example, Li et al. (2022) found that *S. pengshuiensis* M. L. Sheh & Z. Y. Liu and *S. lamelligera* Hance were similar in overall morphology and thus treated the former as a synonymy of the latter. Furthermore, the misidentification of species and misuse of species names occurred frequently due to the various morphological features within species, such as *S. chinensis* Bunge and *S. orthacantha* S. Moore, as well as *S. caerulescens* Franch. and *S. lamelligera* Hance (Chen, 2019), which made it difficult to identify species accurately. Therefore, the revisions for species of this genus, traditionally recognized by morphological features, are necessary and urgent.

A robust phylogenetic framework could provide a valuable information to aid the taxonomic revision of *Sanicula*. In most angiosperms, plastids are usually considered to be inherited from the maternal parent and have low nucleotide substitution rates (Wicke et al., 2011; Wataru and Tsuneaki, 2023). Thus, the plastid genomes (plastomes) have been widely and successfully used for plant phylogenetic analyses (Duminil et al., 2012; Miller et al., 2014; Razafimandimbison et al., 2014; Zhang et al., 2018; Schneider et al., 2021; Xu and Hong., 2021; Ji et al., 2022; Scatigna et al., 2022; Xiang et al., 2022; Baldwin et al., 2023; Fu et al., 2023), especially for those taxonomically controversial taxa within the family Apiaceae (Gou et al., 2020; Ren et al., 2020, 2022; Cai et al., 2022; Liu et al., 2022; Guo et al., 2023; Liu et  al., 2023a; Lei et al., 2022; Gui et al., 2023; Peng et al., 2023; Qin et al., 2023; Song et al., 2023; Tian et al., 2023; Song et al., 2024). For example, Song et al. (2023) transferred *Peucedanum franchetii* C.Y.Wu & F.T.Pu under the genus *Ligusticopsis* Leute based on phylogenetic analysis of ten plastomes. Gui et al. (2023) investigated the divergence and morphological evolution of alpine *Tongoloa* H. Wolff using 27 plastomes and nuclear ribosomal DNA (nrDNA). Guo et al. (2023) reinterpreted the phylogenetic position and taxonomic revision of the genus *Pterocyclus* Klotzsch (Apiaceae, Apioideae) based on 105 complete plastomes, combined with nrITS and morphological evidence. Therefore, plastomes also provided a promising window for studying the genus *Sanicula.* In the previously published studies (Yang et al., 2022; Li et al., 2023; Song et al., 2024), researchers have used the plastomes data to investigate the phylogenetic positions of *Sanicula* members, which has significantly improved our understanding of this taxonomically confused group. However, sampling of this genus was limited and the interspecific relationships of some members were still unclear, such as *S. giraldii* H. Wolff and *S. giraldii* var. *ovicalycina* R. H. Shan & S. L. Liou, *S. tienmuensis* R. H. Shan & Constance and *S. tienmuensis* var. *pauciflora* R. H. Shan & F. T. Pu, and *S. orthacantha* S. Moore and *S. orthacantha* var. *stolonifera* R. H. Shan & S. L. Liou.

**Figure 1:**
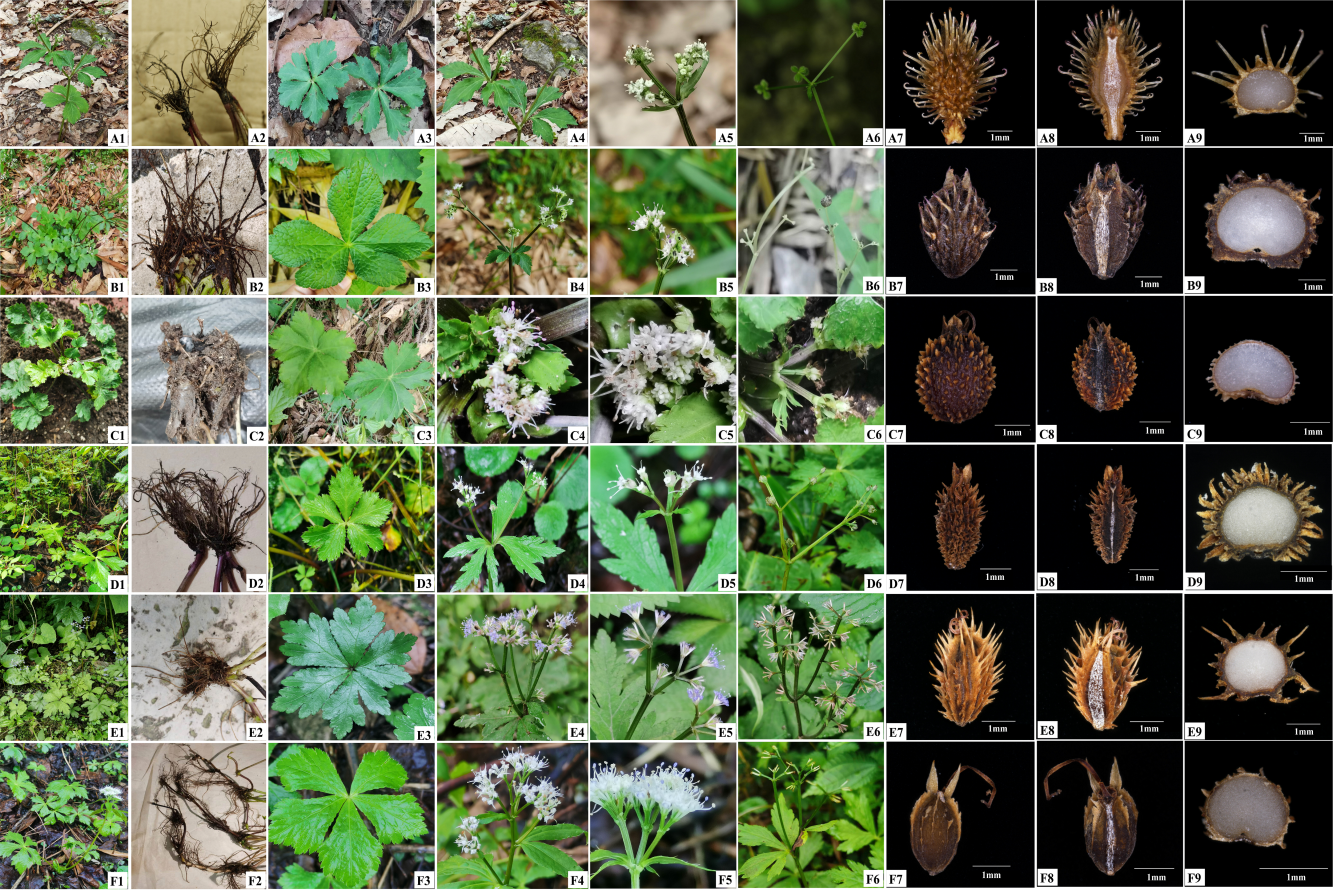
Illustrations of three varieties and three type varieties. **(A)** *S. giraldii*; **(B)** *S. giraldii* var. *ovicalycina*; **(C)** *S. tienmuensis*; **(D)** *S. tienmuensis* var. *pauciflora*; **(E)** *S. orthacantha*; **(F)** *S. orthacantha* var. *stolonifera.* 1. Plant. 2. Root. 3. Basal leaves. 4–5. Flower. 6. Fruit. 7. Dorsal side views of fruits. 8. Commissural side views of fruits. 9. Transverse section.

Therefore, this study mainly focused on three *Sanicula* varieties: *S. giraldii* var. *ovicalycina*, *S. tienmuensis* var. *pauciflora*, and *S. orthacantha* var. *stolonifera*. These three varieties are endemic to China. Shan (1943) described a new species (*S. subgiraldii* R. H. Shan) of the genus. Later, Shan and Liou (1979) also described a new species in Nanchuan, Chongqing, China, which grows on shady woods or grassy places on mountain slopes at an altitude of 1,300 m–1,600 m. They observed that this species was very similar to *S. giraldii*, but the fertile flowers of the species were usually fewer than in *S. giraldii* (one to three per umbellule vs. three per umbellule), with characteristics of broadly ovate calyx teeth, larger size, and oblong fruit, hence they regarded this species as a variety of *S. giraldii* (**Figure 1A**) and named it as *S. giraldii* var. *ovicalycina* (**Figure 1B**). Pimenov (2017) treated *S. subgiraldii* as a synonym of *S. giraldii* var. *ovicalycina* based on reviews of the type specimens and morphological evidence. So far, this variety name was accepted and all authors agreed with this treatment by Shan and Liou (1979). The other variety is *S*. *tienmuensis* var. *pauciflora* described by Shan and Pu (1989). This variety was a narrowly circumscribed species, only occurring in Luding, Sichuan, China. It grows on the edge of ditches or under the forest with an altitude of 2,200 m. *Sanicula tienmuensis* var. *pauciflora* (**Figure 1D**) is considered to be a variety of *S. tienmuensis* (**Figure 1C**), mainly because it has fewer staminate flowers (two or three per umbellule), whereas *S*. *tienmuensis* has more staminate flowers (five or six per umbellule) (Shan and Pu, 1989). The remaining one variety is *S. orthacantha* var. *stolonifera* (**Figure 1F**), which was described as a variety of *S. orthacantha* S. Moore (**Figure 1E**) (Shan and Liou, 1979). This variety grows on mountain top with an altitude of 2,300 m–2,450 m in Emei Shan, Sichuan, China. It can be distinguished from *S. orthacantha* by its thin rhizome and elongate stoloniferous nodes (vs. thick, oblique rootstock bearing elongated, fibrous roots) and ovate calyx teeth, ca. 1 mm long and 0.5 mm wide (vs. narrowly lanceolate, acute, ca. 1 mm long, 0.1 mm wide) (Li and Song, 2022). Sheh and Phillippe (2005) also recognized this variety and stated that the rhizome with long and distinct nodes was its distinctive character. However, after critical examination of type specimen and careful observation in the field, we found that these three varieties were not similar to their type varieties, especially in fruit morphology (**Figure 1**). Therefore, we suggested that the taxonomic positions of *S. giraldii* var. *ovicalycina*, *S*. *tienmuensis* var. *pauciflora*, and *S. orthacantha* var. *stolonifera* need to be re-evaluated.

**Figure 2:**
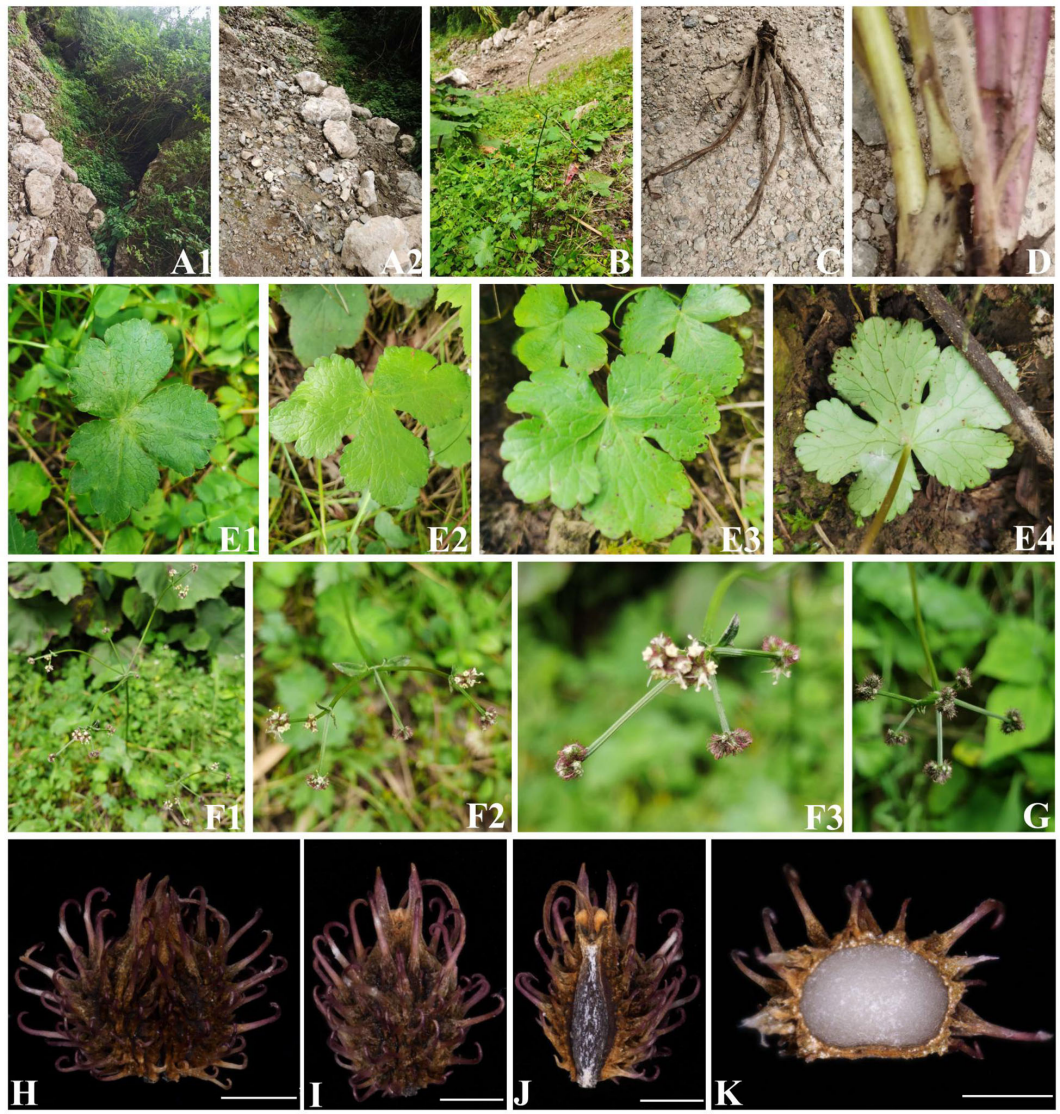
The morphological characters of *Sanicula sp.* SBN2022073001. **(A)** Habit. **(B)** Plant. **(C)** Root. **(D)** Stem. **(E)** Basal leaves. **(F)** Inflorescence and flower. **(G)** Fruit. **(H)** Cremocarp. **(I)** Dorsal side views of fruits. **(J)** Commissural side views of fruits. **(K)** Transverse section. Scale bars: 0.5 mm **(H–K)**.

**Figure 3:**
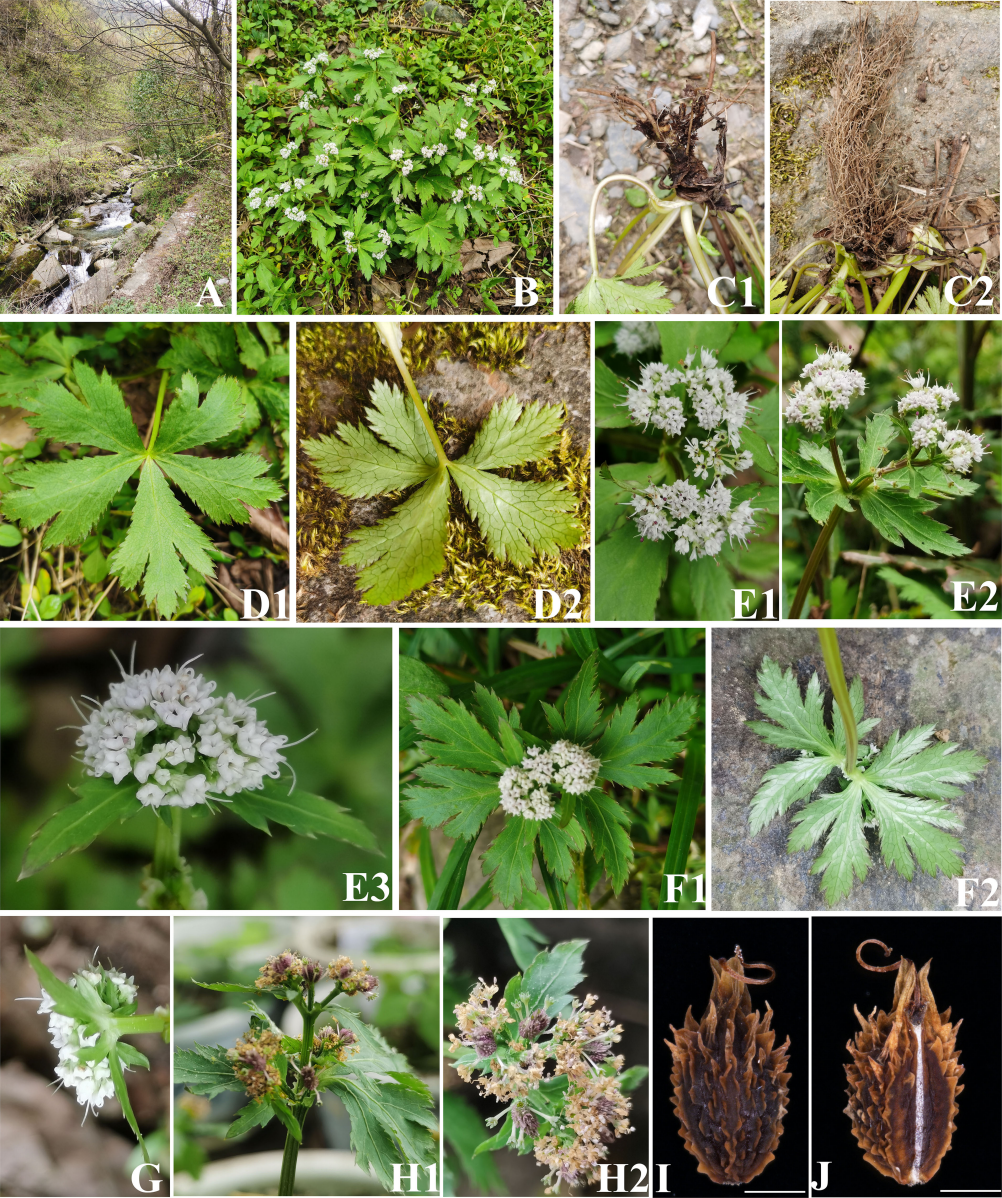
The morphological characters of *Sanicula sp.* SBN2023041201. **(A)** Habit. **(B)** Plant. **(C)** Root. **(D)** Basal leaves. **(E)** Inflorescence and flower. **(F)** Bracts. **(G)** Bracteoles. **(H)** Fruit. **(I)** Dorsal side views of fruits. **(J)** Commissural side views of fruits. Scale bars: 0.5 mm **(I, J)**.

In addition, during two field botanical surveys of Apiaceae in July to September 2022 and March to June 2023, we (I and my colleagues Chang-Kun Liu, Ting Ren, Yu-Lin Xiao) collected two interesting *Sanicula* species: *Sanicula* s*p.* SBN2022073001 (**Figure 2**) and *Sanicula* s*p.* SBN2023041201 (**Figure 3**) in Hanyuan Country, Sichuan Province, and Langao County, Shanxi Province, respectively. *Sanicula sp.* SBN2022073001 grows under the mixed forest or roadsides at an altitude of 2,000 m–2,100 m. *Sanicula sp.* SBN2023041201 grows in stream banks in mixed forests with an altitude of 1,400–1,500 m. By consulting a large number of specimens and investigating the morphological and anatomical characters, we found that both exhibited distinctly different morphological characters with other species of this genus, including distinct differences in leaves, inflorescence, peduncle, bracts, bracteoles, fruit, and calyx teeth. Based on the combination of detailed morphological features and molecular evidence, we confirmed that these two new species actually represented two hitherto undescribed species of *Sanicula*.

In this study, we aim to (1) reveal the plastome features of three varieties and the two undescribed species of *Sanicula*; (2) uncover the phylogenetic positions of these three varieties and the two undescribed species; and (3) provide a taxonomic revision for these three varieties and accept two new members of the genus based on comparative plastome analyses, molecular phylogeny and morphological features.

## 2 Materials and methods

### 2.1 Sample collection, DNA extraction, sequencing, assembly, and annotation

In this study, we collected sixteen individuals of nine *Sanicula* species in the wild and the fresh young basal leaves were immediately dried and stored with silica gel. All voucher specimens were deposited in the Sichuan University Herbarium (SZ) (Chengdu, China) (**Supplementary Table 1**). Herbarium codes are based on Index Herbariorum (https://sweetgum.nybg.org/science/ih/). We used the modified CTAB method (Pahlich and Gerlitz, 1980) to extract total genomic DNA from silica gel-dried leaves, which was then used for subsequent sequencing.

Before library preparation, we used agarose gel electrophoresis to test the quality and quantity of genomic DNA. Then, the DNA library with an average insert size of 400 bp was constructed using the TruSeq DNA Sample Preparation Kits (Illumina) referred to the manufacturer’s protocol (Illumina, San Diego, CA, USA). The DNA library was sequenced using the Illumina NovaSeq platform at Shanghai Personal Biotechnology Co., Ltd. (Shanghai, China), with an average paired-end read length of 150 bp. At least 5 GB of raw data per species was generated. To obtain high-quality reads, the software fastP v0.15.0 (-n 10, -q 15) (Chen et al., 2018) was used to filter raw data. For the yielded high-quality reads, we employed two methods to assemble the complete plastomes. First, the GetOrganelle pipeline (Jin et al., 2020) was used to assemble the sixteen complete plastomes of nine *Sanicula* taxa, using the plastome sequence of *S. giraldii* (OQ612643) as a reference. To validate the accuracy of plastome assembly, we also assembled the sixteen plastomes using the NOVOPlasty v2.6.2 program (Dierckxsens et al., 2017), setting the *rbc*L sequence extracted from the plastome of *S. giraldii* (OQ612643) as the seed. The complete plastomes were initially annotated by Plastid Genome Annotator (PGA) (Qu et al., 2019) software, with *S. giraldii* (OQ612643) as a reference, and then manually checked and corrected the start and stop codons and intron positions in Geneious v9.0.2 (Kearse et al., 2012). Finally, the well-annotated plastomes were displayed by online program OrganellarGenomeDRAW (OGDRAW) (Lohse et al., 2007).

In addition, total DNA was also used to amplify the complete Internal Transcribed Spacers (ITS) region. We employed a 30 μL amplification system, which included 2 µL extracted total DNA, 10 µL ddH_2_O, 15 µL Taq MasterMix (CWBio, Beijing, China), 1.5 µL of 10 pmol µL^−1^ forward primers (ITS-4: 5′-TCC TCC GCT TAT TGA TAT GC-3′), and 1.5 µL of 10 pmol µL ^−1^ reverse primers (ITS-5: 5′-GGA AGT AAA AGT CGT AAC AAG G-3′). The PCR program of ITS started with an initial denaturation step at 94°C for 3 min, followed by denaturation step at 94°C for 45 s, 30 cycles of 45 s at 94°C, annealing at 55°C for 45 s and extension at 72°C for 45 s, a final extension for 7 min at 72°C, and storage at 4°C (White et al., 1990). Then, PCR products were sent to Sangon (Shanghai, China) for sequencing. The software Geneious v9.0.2 (Kearse et al., 2012) was used to assemble and edit the newly generated ITS sequences and gained consensus sequences. Finally, the sixteen newly sequenced plastome data and 43 newly ITS sequences were uploaded in NCBI with the GenBank Accession (OR865876-OR865891) and (OR879918-OR879960), respectively (**Supplementary Table 1**).

### 2.2 Repeat sequence and codon usage

REPuter (Kurtz et al., 2001) was employed to investigate the repeats that included four types: Palindromic (P), Forward (F), Reverse (R), and Complementary (C) repeats. We focused on the repeats with a minimal size of 30 bp, 90% similarity between the two repeat copies, and hamming distance of 3. Moreover, Perl script MISA (http://pgrc.ipk-gatersleben.de/misa/) was used to discover simple sequence repeats (SSRs) in the 21 *Sanicula* plastomes. Moreover, the minimum number of repeat units was set to 10, 5, 4, 3, 3, and 3, for mono-, di-, tri-, tetra-, penta-, and hexanucleotides, respectively.

For codon usage analyses, we extracted the coding sequence (CDS) from 21 *Sanicula* plastomes and deleted duplicates. To avoid sampling bias, we isolated CDSs longer than 300 bp and finally screened 53 CDSs. Then, these 53 CDSs were concatenated by the software Geneious v9.0.2 (Kearse et al., 2012) and the codon bias for each species of *Sanicula* was analyzed using the CodonW v1.4.2 program (Peden, 1999). Finally, the heatmap of the results were drawn using R packages “pheatmap” (https://cran.r-project.org/web/packages/pheatmap/index.html).

### 2.3 Comparative plastome analyses

We compared the IR length and gene location at the IR/SC boundaries among the 21 *Sanicula* plastomes in Geneious v9.0.2 (Kearse et al., 2012). Then, we detected the possible gene rearrangements using the whole genome alignment tool Mauve v1.1.3 plugin (Darling et al., 2004) in Geneious v9.0.2 (Kearse et al., 2012). In addition, we evaluated the degree of variation sequences of these 21 *Sanicula* plastomes using the LAGAN model implemented in the mVISTA (Frazer et al., 2004) tool with default parameters, setting *S. astrantiifolia* as the reference. Finally, to further investigate the hypervariable regions, the protein-coding genes, the non-coding regions, and the intergenic regions among the 21 *Sanicula* plastomes were extracted in Geneious v9.0.2 (Kearse et al., 2012) and aligned with MAFFT v7.221 (Katoh and Standley, 2013). The alignments with less than 200 bp in length were discarded, and then we calculated the nucleotide diversity (Pi) employing DnaSP v5.0 (Librado and Rozas, 2009).

### 2.4 Phylogenetic analyses

We performed the phylogenetic analyses using two datasets: dataset 1 was the 60 complete plastomes (16 newly sequenced) and dataset 2 included 73 ITS sequences (43 newly sequenced and assembled) (**Supplementary Tables 1**, **2**). Among them, *Hedera* L. species were served as the outgroup referring to a previous study (Song et al., 2024). Sequences from the two datasets were respectively aligned using MAFFT v7.221 (Katoh and Standley, 2013) and adjusted manually when necessary. Both identified matrixes were subjected to Maximum-Likelihood (ML) analyses and Bayesian Inference (BI). For ML analyses, RAxML v8.2.8 (Stamatakis, 2014) was performed to reconstruct the phylogenetic trees and estimate the support value (BS) for each node with 1,000 rapid bootstrap replicates and the GTRGAMMA model referring to the RAxML manual. BI analyses were carried out using MrBayes v3.1.2 (Ronquist and Huelsenbeck, 2012), and the best-fitting evolutionary model (GTR+I+G) for plastome data and (GTR+G) for ITS sequences were determined by Modeltest 3.7 (Posada and Crandall, 1998) based on the Akaike information criterion (AIC). Two independent Markov chain Monte Carlo (MCMC) runs of 10 million generations were performed with sampling every 1,000 generations. When the average standard deviation of the splitting frequency fell below 0.01, the MCMC running finished. The initial 25% of trees was discarded as burn-in, and the remaining trees were used to generate the consensus tree and calculate posterior probabilities (PP). Finally, the phylogenetic trees were edited and displayed in FigTree v1.4.2 (Rambaut and Drummond, 2015).

### 2.5 Morphological observations

The fruit characteristics, as one of the most important morphological characters in the classification system of the Apiaceae, have been widely used in taxonomic studies of many genera of Apiaceae (Xiao et al., 2021; Xu et al., 2021; Cai et al., 2022; Lei et al., 2022; Qin et al., 2023). In the present study, we collected mature fruits from eight taxa (three varieties and three type varieties, and two new species) of *Sanicula* in the field and fixed them in formaldehyde–acetic acid–ethanol (FAA) solution. There were thirty representative fruit samples for each species (ten individuals from each species, each with three fruits) selected to observe their morphological characters, and then their overall structure and anatomy were photographed using a stereo microscope (SMZ25, Nikon Corp., Tokyo, Japan). The software MATO (Liu et al., 2023b) was used to measure the thirty representative fruit samples for each species, and then the average value was calculated. The terminology followed the reported study (Kljuykov et al., 2004). Moreover, we also observed other morphological characters based on extensive documentation, specimens information, and fieldwork.

## 3 Results

### 3.1 Plastome features

In this study, we comprehensively compared the whole plastomes of 21 *Sanicula* taxa. The results showed that the size of 21 *Sanicula* plastomes ranged from 154,500 bp (*S. odorata* (Raf.) Pryer & Phillippe) to 155,792 bp (*S. giraldii* var. *ovicalycina*) (**Supplementary Table 3**). All of them possessed a typical quadripartite structure, including a large single-copy region (LSC: 85,074 bp–86,218 bp), a small single-copy region (SSC: 17,049 bp–17,118 bp), and a pair of inverted repeat regions (IRs: 26,176 bp–26,334 bp) (**Supplementary Figure 1**, **Supplementary Table 3**). The total GC content of the 21 *Sanicula* plastomes was 38.1%–38.2%, and the GC content in the LSC, SSC, and IR regions was 36.4%–36.5%, 32.4%–32.6%, and 42.9%–43.0%, respectively (**Supplementary Table 3**). There were 113 unique genes, including 79 protein-coding genes, 30 tRNA genes, and four rRNA genes in the 21 *Sanicula* plastomes (**Supplementary Table 4**).

We investigated the repeat sequences of the 21 *Sanicula* plastomes and detected a total of 977 repeats of four types, containing 482 forward repeats, 478 palindromic repeats, 15 reverse repeats, and two complementary repeats (**Supplementary Figure 2A**, **Supplementary Table 5**). All *Sanicula* plastomes possessed forward and palindromic repeats, twelve taxa had the reverse repeats, and the complementary repeats only occurred in *Sanicula sp.* SBN2023041201 and *S. odorata* (**Supplementary Figure 2A**). In addition, six types of SSRs (mono-, di-, tri-, tetra-, penta-, and hexanucleotide) were identified in the 21 *Sanicula* plastomes (**Supplementary Figure 2B**, **Supplementary Table 5**). The total number of SSRs was 1215, of which the most predominant SSR was mononucleotide (575) and the fewest SSRs were pentanucleotide (2). The number of SSRs also differed among the 21 *Sanicula* plastomes, with *S. rugulosa* Diels owing the fewest (54 SSRs) and *S. odorata* owing the most (64 SSRs) (**Supplementary Figure 2B**). It was noteworthy that all *Sanicula* species detected mononucleotide-to-tetranucleotide SSRs. Pentanucleotide SSRs were only found in *S. flavovirens* Z.H.Chen, D.D. Ma & W. Y. Xie, and hexanucleotide SSRs were only found in *S. hacquetioides* Franch., *S. rubriflora* F. Schmidt, and *S. rugulosa* Diels (**Supplementary Figure 2B**). Bases A and T occurred more frequently than bases G and C in all identified SSRs of the 21 *Sanicula* plastomes (**Supplementary Table 5**).

The 53 CDSs shared by the 21 *Sanicula* plastomes were extracted and connected to analyze the codon usage patterns. These sequences harbored 21,103–21,205 codons, and the codon usage bias was similar across all *Sanicula* plastomes (**Supplementary Table 6**). The highest number of codons were used to encode the Leucine, and the least number of codons were used to encode the Cysteine. We also found that the relative synonymous codon usage (RSCU) values of all codons varied from 0.34 to 1.92 in the 21 *Sanicula* plastomes. Specifically, thirty codons were used frequently with RSCU greater than 1.00 (**Supplementary Figure 3**).

### 3.2 Plastome comparison

The length of the IR region among the 21 *Sanicula* plastomes ranged from 26,176 bp (*S. odorata*) to 26,334 bp (*S. rugulosa*) (**Supplementary Table 3**), and the genes *rps*19, *rpl*2, *trn*H, *trn*N, *ndh*F, and *ycf*1 were located at the junctions of the IR/SC boundaries (**Supplementary Figure 4**). The results showed that 21 *Sanicula* plastomes were conserved in terms of the gene order and gene content at the IR/SC borders (**Supplementary Figure 4**). In detail, the *rps*19 gene, crossing the IRa/LSC boundaries, were located at the LSC and IRa regions with 221 bp and 58 bp. The IRa/SSC borders were located between *trn*N gene and *ndh*F gene, with 2146 bp–2164 bp and 5 bp–11 bp away from the IRa/LSC borders. The borders of IRb/SSC were crossed by the *ycf*1 gene with 3,447 bp–3,479 bp in the SSC region and 1,819 bp–1,837 bp in the IRb region. In the IRb/LSC borders, all the junctions were within the genes between *rpl*2 and *trn*H with 115 bp–118 bp and 2 bp away from the IRb/LSC borders (**Supplementary Figure 4**). Mauve alignment results demonstrated that the gene order of 21 *Sanicula* plastomes were extremely conservative and no rearrangement occurred in gene organization (**Supplementary Figure 5**). The mVISTA program characterized genome divergence, and the result showed that the whole plastome sequences shared high similarity among the 21 *Sanicula* taxa (**Supplementary Figure 6**).

**Figure 4:**
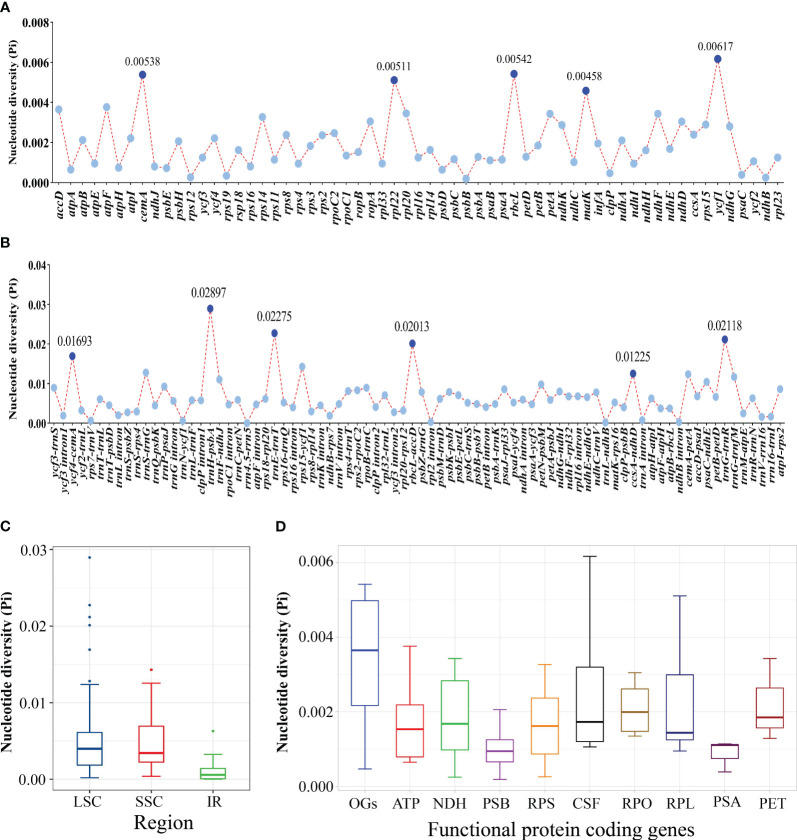
Comparative analysis of the nucleotide diversity (Pi) values among the 21 *Sanicula* plastomes. **(A)** Protein-coding genes. **(B)** Non-coding and intron regions. **(C)** The nucleotide diversity (Pi) in chloroplast regions (IR/SSC/LSC). **(D)** The nucleotide diversity (Pi) of different functional groups.

According to the sequence divergences, eleven mutation hotspot regions were selected as promising DNA barcodes, including five coding regions—*cem*A, *rpl*22, *rbc*L, *mat*K, and *ycf*1—which showed the Pi > 0.00458 (**Figure 4A**, **Supplementary Table 7**) and six non-coding regions—*ycf*4-*cem*A, *trn*H-*psb*A, *trn*E-*trn*T, *rbc*L-*acc*D, *ccs*A-*ndh*D, and *trn*G-*trn*R—which showed the Pi >0.01225 (**Figure 4B**, **Supplementary Table 7**). Meanwhile, the average Pi in the SSC region was higher than that in the IR region (**Figure 4C**). We further found that the other genes (OGs) groups had a higher Pi median value among the functional groups of all protein-coding genes, whereas genes associated with ATP synthase (ATP), photosystems I (PSA), and photosystems II (PSB) had lower Pi median value (**Figure 4D**).

### 3.3 Phylogenetic analyses

**Figure 5:**
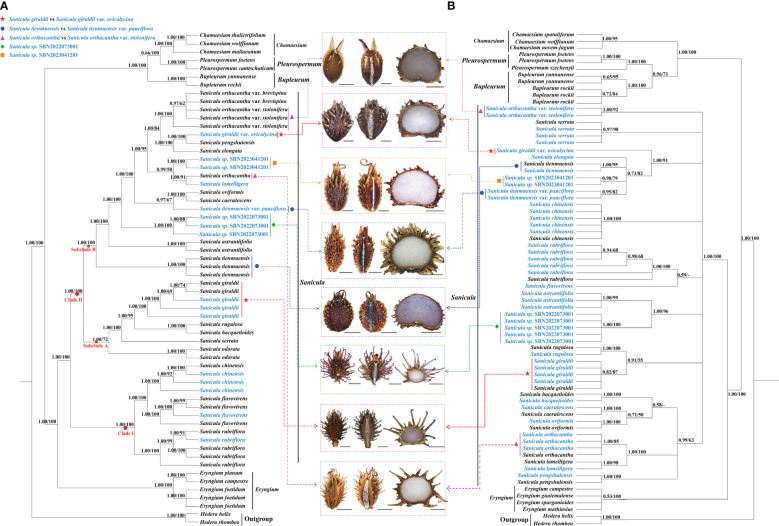
Phylogenetic trees constructed by maximum likelihood (ML) and Bayesian inference (BI). The bootstrap values (BS) of ML and posterior probabilities (PP) of BI are listed at each node. (*) represents the node with PP = 1.00/BS = 100.–means the values < 0.50/50. Light-blue words indicates the newly sequenced species. **(A)**: Plastome tree; **(B)**: ITS tree.

The length of the alignment matrix for the trimmed plastome dataset was 161,260 bp, and the length of the ITS sequence matrix was 593 bp. In our phylogenetic analyses (**Figure 5**, **Supplementary Figure 7**), although several conflicts existed between the plastome phylogenetic tree and the ITS phylogenetic tree, such as *Sanicula sp.*, SBN2022073001 solely formed a clade in the plastome tree (**Figure 5A**), whereas it was sister to *S. astrantiifolia* in the ITS tree (**Figure 5B**), as well as *Sanicula sp.* SBN2023041201 was resolved as sister to *S. orthacantha + S. lamelligera* in the plastome phylogenetic tree (**Figure 5A**), whereas it formed a clade with *S. elongata*, *S. tienmuensis*, and *S. tienmuensis* var. *pauciflora* in the ITS phylogenetic trees (**Figure 5B**); both strongly supported that all *Sanicula* species involved in the current study were well clustered together. The phylogenetic trees also showed that three varieties of our main focus, *S. giraldii* var. *ovicalycina*, *S. tienmuensis* var. *pauciflora*, and *S. orthacantha* var. *stolonifera*, were clearly distant from *S. giraldii*, *S. tienmuensis*, and *S. orthacantha.* In addition, the accessions of *Sanicula sp.* SBN2022073001 and *Sanicula sp.* SBN2023041201 formed their own clades in both trees (**Figure 5**, **Supplementary Figure 7**).

For plastome trees, the phylogenetic topologies of ML and BI analyses were highly identical (**Figure 5A**). The 21 *Sanicula* members scattered in two clades: clade I included three species (*S. rubriflora*, *S. flavovirens*, *S. chinensis*) (PP = 1.00, BS = 100), and the remainders were placed in clade II (PP = 1.00, BS = 100) (**Figure 5A**). In clade II, eighteen *Sanicula* taxa were divided into two subclades (PP = 1.00, BS = 100). Among them, *S. giraldii* was located in subclade A and was sister to *S. rugulosa* + *S. hacquetioides*, whereas *S. giraldii* var. *ovicalycina* nested in subclade B and formed a clade with *S. pengshuiensis* (PP = 1.00, BS = 100). *Sanicula tienmuensis* failed to gather with *S. tienmuensis* var. *pauciflora* but formed a separate clade (PP = 1.00, BS = 100). Instead, *S. tienmuensis* var. *pauciflora* was sister to *S. oviformis* X. T. Liu & Z. Y. Liu + *S. caerulescens* Franch. (PP = 0.97, BS = 67). *Sanicula orthacantha* was more closely related to the *S. lamelligera* (PP = 1.00, BS = 91), whereas *S. orthacantha* var. *stolonifera* clustered with *S. orthacantha* var. *brevispina* H. Boissieu and both formed a separate clade with strong support (PP = 1.00, BS = 100) and was far from *S. orthacantha*. As for the two new species, *Sanicula sp.* SBN2022073001 formed an individually monophyletic clade (PP = 1.00, BS = 100) and *Sanicula sp.* SBN2023041201 made a sister to *S. orthacantha* + *S. lamelligera* (PP = 0.99, BS = 58) (**Figure 5A**).

The analyses of ML and BI based on ITS sequences also yielded consistent tree topologies (**Figure 5B**). Although the phylogenetic trees have low supports and resolutions, the results also indicated that *S. giraldii* var. *ovicalycina*, *S. tienmuensis* var. *pauciflora*, and *S. orthacantha* var. *stolonifera* were also clearly distant from *S. giraldii*, *S. tienmuensis*, and *S. orthacantha.* In detail, *S. giraldii* was resolved as sister to *S. rugulosa* (PP = 0.91, BS = 55), whereas *S. giraldii* var. *ovicalycina* solely represented a clade with high support (PP = 1.00, BS = 91). *Sanicula tienmuensis*, *S. tienmuensis* var. *pauciflora*, *S. elongata*, and *Sanicula sp.* SBN2023041201 clustered a clade (PP = 0.73, BS = 82). *Sanicula orthacantha* still had close affinity to *S. lamelligera* (PP = 1.00, BS = 100), whereas *S. orthacantha* var. *stolonifera* formed a separate clade and was distant from *S. orthacantha.* For the two new species, *Sanicula sp.* SBN2022073001 was sister to *S. astrantiifolia* (PP = 1.00, BS = 96), and *Sanicula sp.* SBN2023041201 formed a clade with *S. tienmuensis*, *S. elongata*, and *S. tienmuensis* var. *pauciflora* (PP = 0.73, BS = 82) (**Figure 5B**).

### 3.4 Morphological characteristics

Fruits (mericarps) of eight *Sanicula* taxa were mapped to the two phylogenetic trees (**Figure 5**). The detailed information of fruit anatomical and micromorphological characteristics of the eight *Sanicula* species were shown in **Table 1**. The other morphological features were also presented in **Supplementary Table 8**.

**Table 1:** Fruit morphological and anatomical characteristics of nine *Sanicula* species.

Taxa	Shape	Fruit surface	Calyx teeth	Cross section	Endosperm on commissural side	Vittae
*S. giraldii*	Narrowly ovoid	Densely covered with developed yellow or purplish red uncinate bristles, long and hard	Ovate and small, tip mucronate	Ellipsoid	Flat	Obscure
*S. giraldii* var. *ovicalycina*	Broadly ovate	Rarely covered with purplish red short bristles, proximal end with tubercles, obscure, distal end with uncinate bristles or straight	Broadly ovate and large	Suborbicular	Slightly concave	Obscure
*S. tienmuensis*	Subglobose	Covered with short and obtuse prickles, slight formed scales and tubercles	Broadly ovate	Reniform	Slightly concave	Obscure
*S. tienmuensis* var. *pauciflora*	Long ellipsoid	Densely covered with sharp prickles	Long-lanceolate	Suborbicular	Flat	Vittae 2 in commissural side
*S. orthacantha*	Narrowly ovoid	Densely covered with short, straight and sharp spines, and sometimes the base formed a thin layer, fruit ribs and furrows spinulose	Narrowly lanceolate	Slightly circular	Flat	Obscure
*S. orthacantha* var. *stolonifera*	Ovoid	Proximal end with degenerated to disappeared the prickles, nearly smooth, distal end with prickles and formed a thin layer	Ovate	Reniform	Slightly concave	Obscure
*S. orthacantha* var. *brevispina*	Oblong ovoid to ovoid	Usually with erose-spinulose ribs and furrows smooth or barely spinulose	Linear to lanceolate	/	/	/
*Sanicula* sp. SBN2022073001	Broadly ovate	Densely covered with purplish red uncinate prickles	Lanceolate	Elliptical	Flat	Obscure
*Sanicula* sp. SBN2023041201	Ellipsoid	Proximal end with scalariform prickles, not acute, distal end with acute prickles	Narrowly ovate	Reniform	Slightly concave	Obscure

The morphological characteristics of *S. orthacantha* var. *brevispina* were based on the study of Li and Song (2022). “/” represented the information was missing.

The key morphological features of *S. giraldii* were the fruit densely covered with developed yellow or purplish red, long and hard uncinate bristles as well as ovate and small calyx teeth, the tip mucronate (**Figures 1A**, **5**, **Table 1**); the inflorescence was 2–4-trichotomously branched. All branches elongate, and the leaf vein surface was smooth (**Figure 1A**, **Supplementary Table 8**). *Sanicula giraldii* var. *ovicalycina* had the unique characteristics of the fruits rarely covered with purplish red short bristles, proximal end with tubercles, obscure, distal end with uncinate bristles or straight, calyx teeth broadly ovate and large (**Figures 1B**, **5**, **Table 1**), inflorescence dichotomously cymose-branched, leaf veins distinctly concave in adaxial surface, distinctly prominent in abaxial surface, gridded (**Figure 1B**, **Supplementary Table 8**).

*Sanicula tienmuensis* had fruits subglobose, covered with short and obtuse prickles, slight formed scales and tubercles, calyx teeth broadly ovate, and vittae obscure (**Figures 1C**, **5**, **Table 1**). *Sanicula tienmuensis* var. *pauciflora* had fruits long ellipsoid, densely covered with sharp prickles, calyx teeth long-lanceolate, vittae 2 in commissural side (**Figures 1D**, **5**, **Table 1**).

The distinctive features of *S. orthacantha* were as follows: the fruit was narrowly ovoid, densely covered with short, straight and sharp spines, and sometimes the base formed a thin layer, fruit ribs and furrows spinulose, narrowly lanceolate calyx teeth (**Figures 1E**, **5**, **Table 1**), the inflorescence was 2-3-branched; umbels 3–8, sometimes 1 shortened branch between forks or on lateral branches, its rootstock short, tuberlike, woody, bearing a fascicle of thinly fibrous roots (**Figure 1E**, **Supplementary Table 8**). *Sanicula orthacantha* var. *stolonifera* had the ovoid fruit with proximal end with degenerated to disappeared the prickles, nearly smooth, whereas distal end with prickles and formed a thin layer, ovate calyx teeth, tip sharp (**Figures 1F**, **5**, **Table 1**), slender, elongate and lignified nodes stoloniferous rhizomes (**Figure 1F**, **Supplementary Table 8**).

For these two new members, *Sanicula sp.* SBN2022073001 had fruits broadly ovate, densely covered with purplish red uncinate prickles, calyx teeth lanceolate, not covered with prickles, vittae obscure (**Figures 2**, **5**, **Table 1**), inflorescence pleiochasium-branched, 3–6, unequal, bracts small or degraded, bracteoles 2, opposite, linear–lanceolate (**Figure 2**, **Supplementary Table 8**). *Sanicula sp.* SBN2023041201 had fruits ellipsoid, proximal end with scalariform prickles, not acute, distal end with acute prickles, calyx teeth narrowly ovate (**Figures 3**, **5**, **Table 1**), inflorescence dichotomously cymose-branched, extremely shortened peduncle, staminate flowers 9–10 per umbellule, usually 9 (**Figure 3**, **Supplementary Table 8**).

## 4 Discussion

### 4.1 Plastome evolution

The 21 *Sanicula* plastomes exhibited a typical quadripartite structure, and they also shared extremely similar genomic size, GC content, IR borders, the patterns of codon bias and SSR, as well as identical gene content and order. These findings showed that the 21 *Sanicula* plastomes were highly conserved. Although the 21 plastomes displayed high similarity, eleven mutation hotspot regions (*cem*A, *rpl*22, *rbc*L, *mat*K, *ycf*1, *ycf*4-*cem*A, *trn*H-*psb*A, *trn*E-*trn*T, *rbc*L-*acc*D, *ccs*A-*ndh*D, and *trn*G-*trn*R) were still identified. Except for three universal DNA barcodes (*rbc*L, *mat*K, *trn*H-*psb*A) (Richardson et al., 2000; Pridgeon et al., 2001; Muellner et al., 2003; Nyffeler et al., 2005; Cássio et al., 2009), the remaining eight fragments could be served as potential DNA barcodes to discriminate those *Sanicula* taxa that were difficult to identify by morphological features, such as *S. chinensis* and *S. orthacantha*, as well as *S. caerulescens* and *S. lamelligera.*

### 4.2 Phylogenetic inference and taxonomic implication

In the present study, we performed phylogenetic analyses using complete plastomes and ITS sequences. Unfortunately, the plastome-based and ITS-based phylogenetic trees yielded incongruent topologies. The phenomenon of conflict was also frequently observed in other genera of Apiaceae (Ren et al., 2020; Cai et al., 2022; Ren et al., 2022; Wen et al., 2021; Gui et al., 2023; Qin et al., 2023; Song et al., 2023; Tian et al., 2023; Song et al., 2024). This conflict was likely attributed to the biparental inheritance, higher mutation rate, and the insufficient sequence length of ITS data, whereas plastid DNA was maternal inheritance and has lower mutation rate (Wolfe et al., 1987; Koch et al., 2001). Moreover, the hybridization/introgression and incomplete lineage sorting (ILS) may be responsible for the inconsistent relationships between plastome-based and ITS-based phylogenies (Wen et al., 2021). Further study is needed to identify the cause of the nuclear-plastome conflict in *Sanicula*. Although the conflicts existed between the plastome tree and ITS tree, both strongly suggested that *S. giraldii* var. *ovicalycina*, *S. tienmuensis* var. *pauciflora*, and *S. orthacantha* var. *stolonifera* were extremely distant from *S. giraldii*, *S. tienmuensis*, and *S. orthacantha*, respectively, which implied that these three varieties should be regarded as three independent species. Moreover, the morphological characteristics of three varieties also supported the above phylogenetic results. Furthermore, we also clarified the species relationships with ambiguous systematic position, such as *S. pengshuiensis* and *S. lamelligera*, and suggested that *S. pengshuiensis* should be regarded as an independent species rather than a synonymy of *S. lamelligera.*

Both phylogenetic analyses (**Figure 5**) showed that *S. giraldii* var. *ovicalycina* was distant from *S. giraldii*, which implied that this variety should not be regarded as a variety but rather as an independent species. Multiple morphological characteristics also further supported the above phylogenetic results (**Figure 1**, **Table 1**, **Supplementary Table 8**). Previously, Shan (1943) described a species (*S. subgiraldii*) of the genus based on the nomenclatural type specimen of *S. giraldii* var. *ovicalycina*. Subsequently, Pimenov (2017) reduced *S. subgiraldii* to the synonym *S. giraldii* var. *ovicalycina* in his checklist of Chinese Umbelliferae. By examination of herbarium specimens and observations on living plants in field, we found that the *S. subgiraldii* and *S. giraldii* var. *ovicalycina* was identical in morphology, such as the smaller primary polyphylla, the longer flowering branches and the basally obsoletely setulous-crenate leaf segments, the fruit was broadly ovate, vittae obscure. Therefore, according to the International Code for Nomenclature for plants, we reinstated the independent specific status of *S. subgiraldii* and suggested that *S. subgiraldii* should be as a legitimately accepted name and treated *S. giraldii* var. *ovicalycina* as a synonym of *S. subgiraldii.*

Li and Song (2022) found that *S. orthacantha* var. *stolonifera* was identical with *S. orthacantha* var. *brevispina* in morphology, especially in the erose-spinulose ribs and spinulose or smooth furrows of the fruits and the number of staminate flowers per umbellule, and thus merged *S. orthacantha* var. *stolonifera* into *S. orthacantha* var. *brevispina*. Our plastome evidence and morphological data also strongly supported this treatment (**Figure 5**, **Table 1**, **Supplementary Table 8**). Moreover, our phylogenetic tree showed that *S. orthacantha* var. *brevispina* clustered together with *S. orthacantha* var. *stolonifera* and formed a separate clade, which was distant from *S. orthacantha.* These findings indicated that *S. orthacantha* var. *brevispina* should also be considered as an independent species, rather than a variety of *S. orthacantha*, which was further verified by morphological evidence (**Figure 1**, **Table 1**, **Supplementary Table 8**). Therefore, treating *S. orthacantha* var. *brevispina* as an independent species was reasonable and convincing, and a new independent species of the genus was presented.

Phylogenetic analyses based on plastome data and ITS sequences showed that *S. tienmuensis* var. *pauciflora* was clearly distant from *S. tienmuensis* (**Figure 5**), implying that the variety should not be regarded as a variety, but rather as an independent species. Moreover, the morphological characteristics also further supported the above phylogenetic results. For example, the key morphological features that distinguished *S. tienmuensis* and *S. tienmuensis* var. *pauciflora* were the calyx teeth and fruit. *S. tienmuensis* had broadly ovate calyx teeth; subglobose fruit, covered with short and obtuse prickles, slight formed scales and tubercles; endosperm slightly concave on commissural side, whereas long-lanceolate calyx teeth; long ovate fruit, densely covered with sharp prickles; flat endosperm on commissural side were existed in *S. tienmuensis* var. *pauciflora* (**Figures 1D, E**, **Table 1**, **Supplementary Table 8**).

In addition, we also investigated these two undescribed species (*Sanicula sp.* SBN2022073001 and *Sanicula sp.* SBN2023041201). Both phylogenetic trees firmly supported that the individuals of *Sanicula sp.* SBN2022073001 gathered together (**Figure 5**). In the plastome tree (**Figure 5A**), *Sanicula sp.* SBN2022073001 solely formed a clade. Although *Sanicula sp.* SBN2022073001 was sister to *S. astrantiifolia* in the ITS tree (**Figure 5B**), it can be discriminated from *S. astrantiifolia* by its unique characters, such as inflorescence pleiochasium-branched, 3–6, unequal, bracts small or degraded, bracteoles 2, opposite, linear-lanceolate, umbellules 4–7-flowered, staminate flowers 3–5 per umbellule, fertile flowers 1–2 per umbellule, pedicels extremely shortened, as long as fertile flowers (**Figure 2**, **Supplementary Table 8**), whereas inflorescence cymose branched, middle branches shorted, bracts 2, linear-lanceolate, bracteoles 7–10, midrib distinct, umbellules ca. 10-flowered, staminate flowers 6–8 per umbellule, pedicels short; petals greenish white or pinkish, fertile flowers 2 or 3 per umbellule, sessile were examined in *S. astrantiifolia* (Shan and Constance, 1951; Sheh and Phillippe, 2005). Therefore, based on molecular phylogenetic analyses and morphological characteristics, we confirmed that *Sanicula sp.* SBN2022073001 was sufficiently different from *S. astrantiifolia* and described it here as a new species, *Sanicula hanyuanensis* B.N.Song, C.K.Liu & X.J.He, *sp. nov.*

The another new species (*Sanicula sp.* SBN2023041201) was resolved as sister to *S. orthacantha + S. lamelligera* in the plastome phylogenetic tree (**Figure 5A**), whereas it formed a clade with *S. elongata*, *S. tienmuensis*, and *S. tienmuensis* var. *pauciflora* in the ITS phylogenetic trees (**Figure 5B**). It noticed that *Sanicula sp.* SBN2023041201 can be discriminated from these five *Sanicula* species by its clearly different morphological characteristics, such as inflorescence dichotomously cymose-branched, extremely shortened peduncle, staminate flowers 9–10 per umbellule, usually 9, calyx teeth narrowly ovate, fruit long ellipsoid, proximal end with scalariform prickles, not acute, distal end with acute prickles (**Figure 3**, **Table 1**, **Supplementary Table 8**). Hence, there is no doubt that *Sanicula sp.* SBN2023041201 was also a new member of *Sanicula* and we described it here as a new species, *Sanicula langaoensis* B.N.Song, T. Ren & X.J.He, *sp. nov.*

### 4.3 Taxonomic treatment

*Sanicula subgiraldii* R.H.Shan.

≡ *Sanicula giraldii* H. Wolff var. *ovicalycina* R.H.Shan & S.L.Liu, in Shan Renhwa & Sheh Menglan (eds.), Fl. Reipubl. Popularis Sin. 55(1): 297,1979.

Type: CHINA. “Szechwan, Nanchuan Hsien, 01. 05. 1930, Chang 277” (holotype NAS!).

Distribution and habitat: This species is endemic to China, growing in hillside meadows or shaded forests with elevations of 1,300 m−1,935 m.

Additional specimens examined: CHINA. Chongqing, 1,600 m alt., 4 July 1983, M.L. Shen 83664 (NAS); Sichuan, 1,500 m alt., 19 May 1964, H.F. Zhou & H.Y. Li 108211 (SZ); Chongqing, 26 May 1957, J.H. Xiong & G.F. Li 90990 (SZ); Chongqing, 26 May 1957, J.H. Xiong 90990 (SZ); Sichuan, 30 June 1964, 90990 (SM); Sichuan, 15 May 1964, M.F. Zhou & S.G. Tang 0055(SM); Chongqing, 1738m alt., 7 July 2022, B.N. Song and C.K. Liu SBN2022070702 (SZ).

*Sanicula pauciflora* (R.H.Shan & F.T.Pu) B.N.Song & X.J.He, comb. et stat. nov.

≡ *Sanicula tienmuensis* R.H.Shan & Constance var. *pauciflora* R.H.Shan & F.T.Pu, Acta Phytotax. Sin. 27(1): 66,1989.

Type: CHINA. Sichuan: Ludung, alt. 2200 m, under forests or by streams, 01 May 1984, Li Yongjiang 115 (holotype CDBI!).

Distribution and habitat: This species is endemic to China (Sichuan, Ludung), occurring in the edge of a ditch or woods in valleys at an elevation of 2,300 m.

Additional specimens examined: CHINA. Ludung, 1 June 1984, Y.L.Cao 115 (CDBI); Ludung, 1998 m alt., 22 June 2022, B.N. Song and Y.L. Xiao SBN2022062201 (SZ).

*Sanicula brevispina* (H. Boissieu) B.N.Song & X.J.He, comb. et stat. nov.

≡ *Sanicula orthacantha* S. Moore var. *brevispina* H. Boissieu, Bull. Soc. Bot. France 53: 421, 1906.

Type: CHINA, Sichuan, Emei Shan, E.H. Wilson 7104 (holotype P! – barcode P03226637; isolectotypes BM!, K!).

= *Sanicula orthacantha* var. *stolonifera* R.H.Shan & S.L.Liu, in Fl. Reipubl. Popularis Sin. 55(1): 53, 297,1979.

Type: CHINA, Sichuan, Emei Shan, Jingangzui, 2450 m, 8 May 1957, K.H. Yang 54432 (lectotype NAS00040551, designated by Li et al., 2022, isolectotype KUN0463177).

Distribution and habitat: This species is endemic to China (Emei Shan, Sichuan), and it grows on slopes or at forest margins at altitude of 1,900 m−2,865 m.

Additional specimens examined: CHINA. Sichuan, 27 July 1960, 13155 (SM); Sichuan, 1935, T.H. Tu 231 (PE); Sichuan, 1935, T.H. Tu 55 (PE); Sichuan, 17 July 1930, W.P. Fang 6527 (NAS); Sichuan, 14 July, 1967, 3906 (SM); Sichuan, 20 August 1930, W.P. Fang 8437 (NAS); Sichuan, 29 July 1962, 7558 (SM); Sichuan, 1450 m alt., 4 June 1957, K.H. Yang 55113 (KUN); Sichuan, 22 June 1940, S.L. Sun 2559 (KUN); Sichuan, 12 August, 1935, Y.Y. Ho 5998 (NAS); CHINA. Sichuan, 9 July 2022, B.N. Song and C.K. Liu SBN2022070901 (SZ).

*Sanicula hanyuanensis* B.N.Song, C.K.Liu & X.J.He, *sp. nov.* (**Figure 2**).

Diagnosis: *Sanicula hanyuanensis* can be identified by the following morphological features such as inflorescence pleiochasium-branched, 3–6, unequal, bracts small or degraded, bracteoles 2, opposite, linear-lanceolate.

Type: CHINA. Sichuan: Hanyuan County, under the mixed forest or roadsides; 29°22′1.33″N, 102°56′10.4″E; elevation 2092 m, 70 July 2022, SBN2022073001 (holotype: SZ) (**Supplementary Figure 8**).

Etymology: The species is named after Hanyuan County, Sichuan Province, China, where it is the type locality.

Description: Perennial herb, plants 40 cm–80 cm high. Taproot short and stout. Stem 1, erect, branched above, green purplish to purple. Basal leaves several; petioles 5 cm–16 cm, blade orbicular, reniform-rounded or broadly cordate, 5–8.5 × 3.5–7 cm, palmately deeply 3-parted to 5-parted; central segment broadly obovate, 2.5–4 × 1.5–4 cm, distally shallowly 3-lobed, base cuneate, apex obtuse-rounded; lateral segments rhombic-rounded or broadly obovate, 2.5–3 × 1.5–2 cm, distally shallowly 3-lobed, primary veins 3–5, prominent on both surfaces. Upper leaves small or degraded. Inflorescence pleiochasium-branched, 3–6, unequal, 3–16 cm, bracts small or degraded, bracteoles 2, 0.6–1 × 0.1–0.4 cm, opposite, linear-lanceolate. Umbellules 4–7-flowered, staminate flowers 3–5 per umbellule, fertile flowers 1–2 per umbellule, pedicels extremely shortened, as long as fertile flowers. Calyx teeth lanceolate, ca. 1 × 0.5 mm; styles ca. 2 mm, recurved. Fruit broadly ovate, densely covered with purplish red uncinate prickles, ca. 4–5 × 3–4 mm, densely covered with purple-red uncinate prickles, vittae obscure. Fl. and fr. Jun–Sep.

Phenology: The flowering and fruiting period is from June to September.

Distribution and habitat: This species is distributed in Hanyuan County, Sichuan Province, China, and grows under the mixed forest or roadsides at an altitude of 2000–2200 m.

*Sanicula langaoensis* B.N.Song, T. Ren & X.J.He, *sp. nov.* (**Figure 3**).

Diagnosis: *Sanicula langaoensis* can be identified by the following morphological features, such as inflorescence dichotomously cymose-branched, extremely shortened peduncle, staminate flowers 9–10 per umbellule, usually 9, calyx teeth narrowly ovate.

Type: CHINA. Shanxi, Langao country, in stream banks in mixed forests; 32°13′47.88″N, 108°53′45.18″E; elevation 1,496 m, 12 April 2023, SBN2023041201 (holotype: SZ) (**Supplementary Figure 9**).

Etymology: The species is named after Langao country, Shanxi Province, China, where it is the type locality.

Description: Perennial herb, plants 15 cm–30 cm high. Rootstock stout, short, fibrous roots brown and numerous. Stems 2–8, erect or oblique, unbranched. Basal leaves numerous; petioles 8–16 (–25) cm, leaf blade subrounded, round-cordate or pentagonal, palmately 3–5-parted, margin sharply irregular-serrate; central segment cuneate-obovate or ovate, 0.8–3.5 × 0.6–2.5 cm; lateral segments parted nearly to base, 1.5–3 × 1–1.5 cm; base cuneate, upper leaves undeveloped. Inflorescence extremely shortened peduncle, ca. 0.5 cm, bracts 2, foliaceous, entire or 2–3-lobed, ca 2 × 1.5 cm; opposite, umbellules 10–11-flowered, staminate flowers 9–10 per umbellule, usually 9, fertile flowers 1 per umbellule. calyx teeth narrowly ovate, ca. 1 × 0.3 mm; styles ca. 2 mm–2.5 mm, recurved. Fruit ellipsoid, ca. 3.5 × 2.5 mm, proximal end with scalariform prickles, not acute, distal end with acute prickles. vittae obscure. Fl. and fr. Mar–Jun.

Phenology: The flowering and fruiting period is from March to June.

Distribution and habitat: This species is distributed in Langao country, Shanxi Province, China, and grows in stream banks in mixed forests at an altitude of 1,400 m–1,660 m.

## Data availability statement

The data presented in the study are deposited in the NCBI repository, accession number OR865876-OR865891 and OR879918-OR879960.

## Author contributions

BS: Investigation, Methodology, Data curation, Writing – original draft, Formal analysis. CL: Methodology, Formal analysis, Writing – original draft. TR: Validation, Methodology, Writing – original draft. YX: Software, Writing – original draft. LC: Methodology, Writing – review & editing. DX: Methodology, Software, Writing – review & editing. AH: Investigation, Writing – review & editing. PX: Investigation, Writing – review & editing. XF: Investigation, Writing – review & editing. SZ: Supervision, Writing – review & editing. XH: Supervision, Writing – review & editing.

## Supplementary material

Supplementary Figure 1 Gene line map of the *Sanicula* plastome. The genes exhibited outside of the line are transcribed clockwise, whereas those are counterclockwise inside. The genes belonging to different functional groups are color-coded.

Supplementary Figure 2 Analyses of repeats in the 21 *Sanicula* plastomes. (A) Total number of four repeat types; (B) Total number of SSRs.

Supplementary Figure 3 The RSCU values of 53 protein coding regions for 21 *Sanicula* plastomes. The red represents higher RSCU values while the blue indicates lower RSCU values.

Supplementary Figure 4 Comparison of the IR/SC borders across the 21 *Sanicula* plastomes. Functional genes and truncated fragments are denoted by colored boxes. The sizes of gene fragments located at boundaries are indicated by the base pair lengths.

Supplementary Figure 5 Mauve alignment of 21 *Sanicula* plastomes. Local collinear blocks within each alignment are represented by blocks of the same color connected with lines.

Supplementary Figure 6 Sequence identity plot comparing the 21 *Sanicula* plastomes using mVISTA. The y-axis corresponds to percentage identity (50–100%), while the x-axis shows the position of each region within the locus. Arrows indicate the transcription of annotated genes in the reference genome.

Supplementary Figure 7 The phylograms constructed by maximum likelihood (ML) and Bayesian inference (BI). The bootstrap values (BS) of ML and posterior probabilities (PP) of BI are listed at each node. (*) represents the node with PP=1.00/BS=100. – means the values < 0.50/50. Light- blue words indicates the newly sequenced species. (A): Plastome tree; (B): ITS tree.

Supplementary Figure 8 Holotype of *Sanicula sp.* SBN2022073001.

Supplementary Figure 9 Holotype of *Sanicula sp.* SBN2023041201.

Supplementary Table 1 Taxa newly sequenced in the present study with source, voucher and GenBank accession numbers.

Supplementary Table 2 Plastomes data and ITS sequences included in phylogenetic analyses with GenBank accession.

Supplementary Table 3 Comparison of plastome features among 21 *Sanicula* plants.

Supplementary Table 4 List of unique genes identified in 21 *Sanicula* plastomes.

Supplementary Table 5 Repeat sequence and Simple Sequence Repeats in 21 *Sanicula* plastomes.

Supplementary Table 6 Codon usage and relative synonymous codon usage (RSCU) values of 53 protein-coding genes of 21 *Sanicula* plastomes.

Supplementary Table 7 Nucleotide diversity (Pi) of coding and non-coding regions.

Supplementary Table 8 The other morphological characteristics of eleven *Sanicula* species.
