# Supplementary figures and images for: Plastid phylogenomics contributes to the taxonomic revision of taxa within the genus Sanicula L. and acceptance of two new members of the genus
Source: Front Plant Sci. 2024 Jun 10;15:1351023. doi: 10.3389/fpls.2024.1351023 (PMC11194442; doi:10.3389/fpls.2024.1351023)

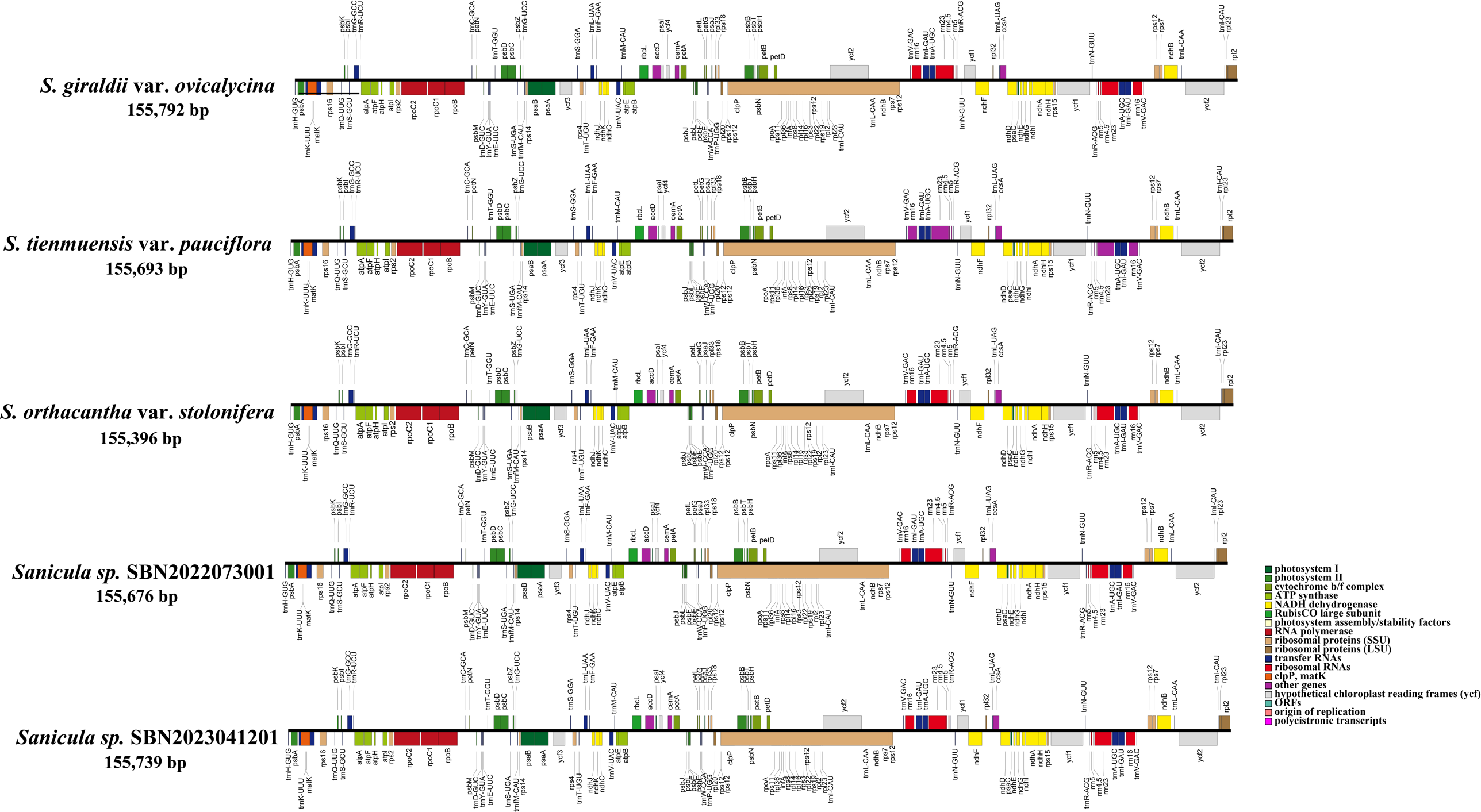

Supplement: Supplementary Figure 1 — Gene line map of the Sanicula plastome. The genes exhibited outside of the line are transcribed clockwise, whereas those are counterclockwise inside. The genes belonging to different functional groups are color-coded. [file Image_1.jpeg]

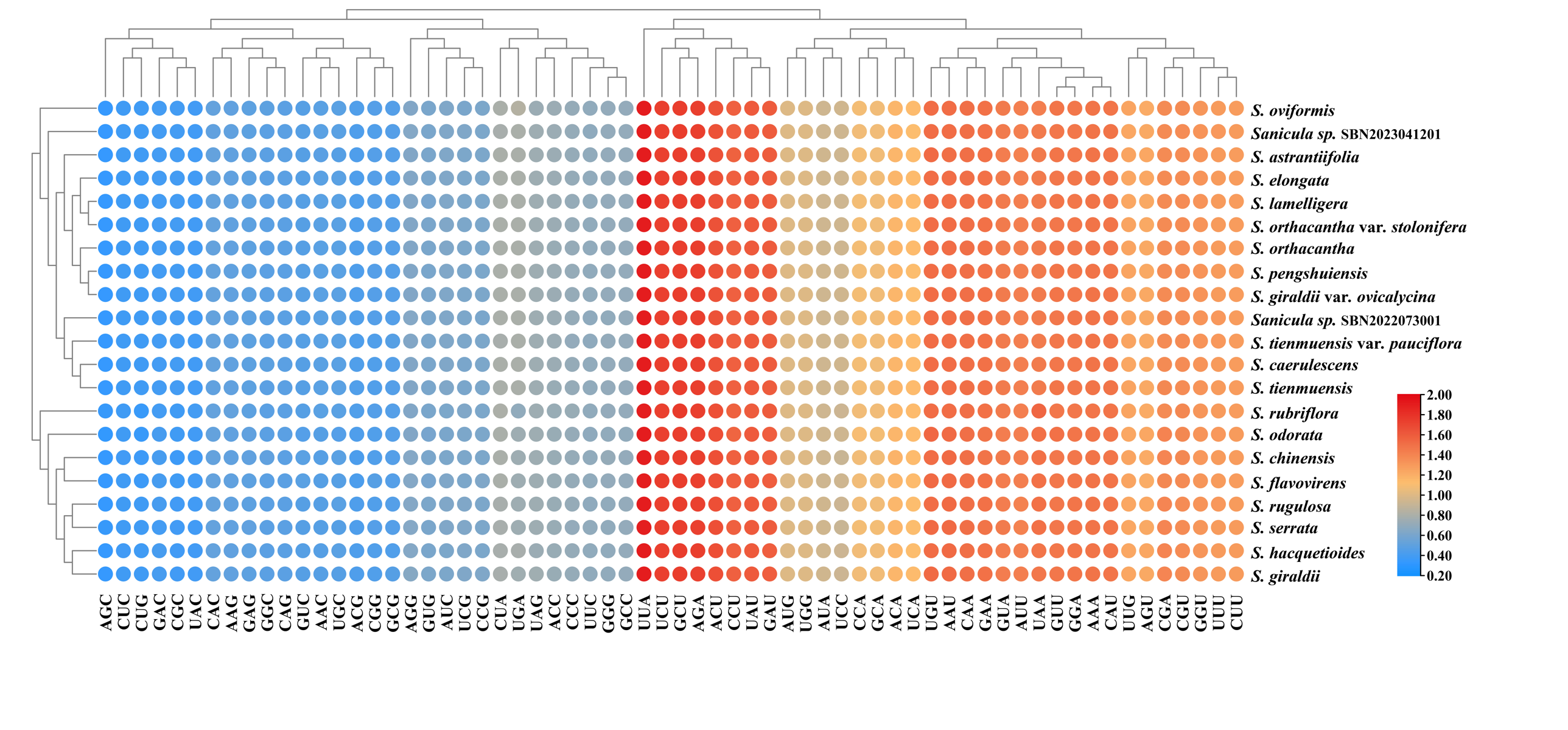

Supplement: Supplementary Figure 3 — The RSCU values of 53 protein coding regions for 21 Sanicula plastomes. The red represents higher RSCU values while the blue indicates lower RSCU values. [file Image_3.jpeg]

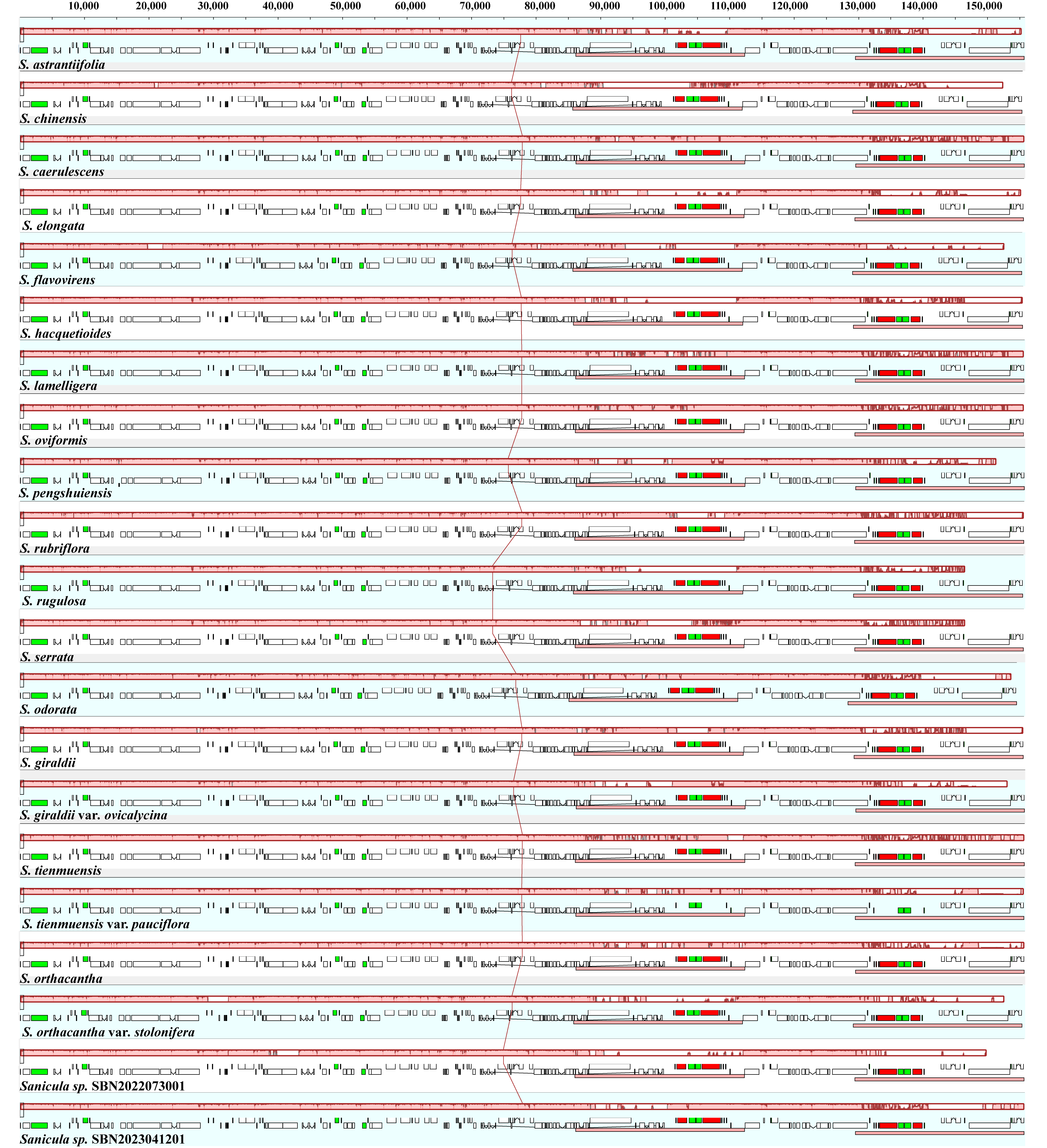

Supplement: Supplementary Figure 5 — Mauve alignment of 21 Sanicula plastomes. Local collinear blocks within each alignment are represented by blocks of the same color connected with lines. [file Image_5.tif]

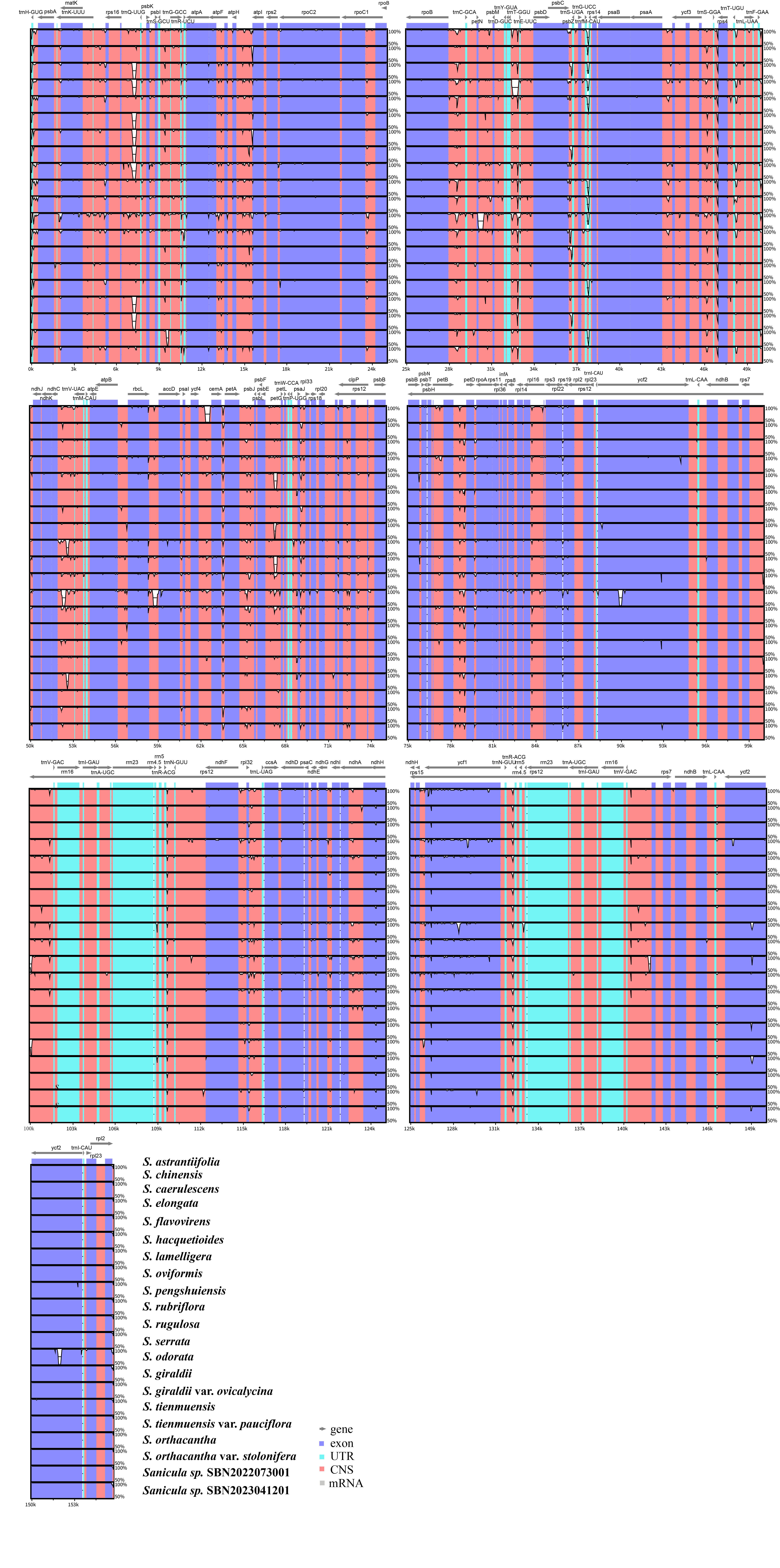

Supplement: Supplementary Figure 6 — Sequence identity plot comparing the 21 Sanicula plastomes using mVISTA. The y-axis corresponds to percentage identity (50–100%), while the x-axis shows the position of each region within the locus. Arrows indicate the transcription of annotated genes in the reference genome. [file Image_6.jpeg]

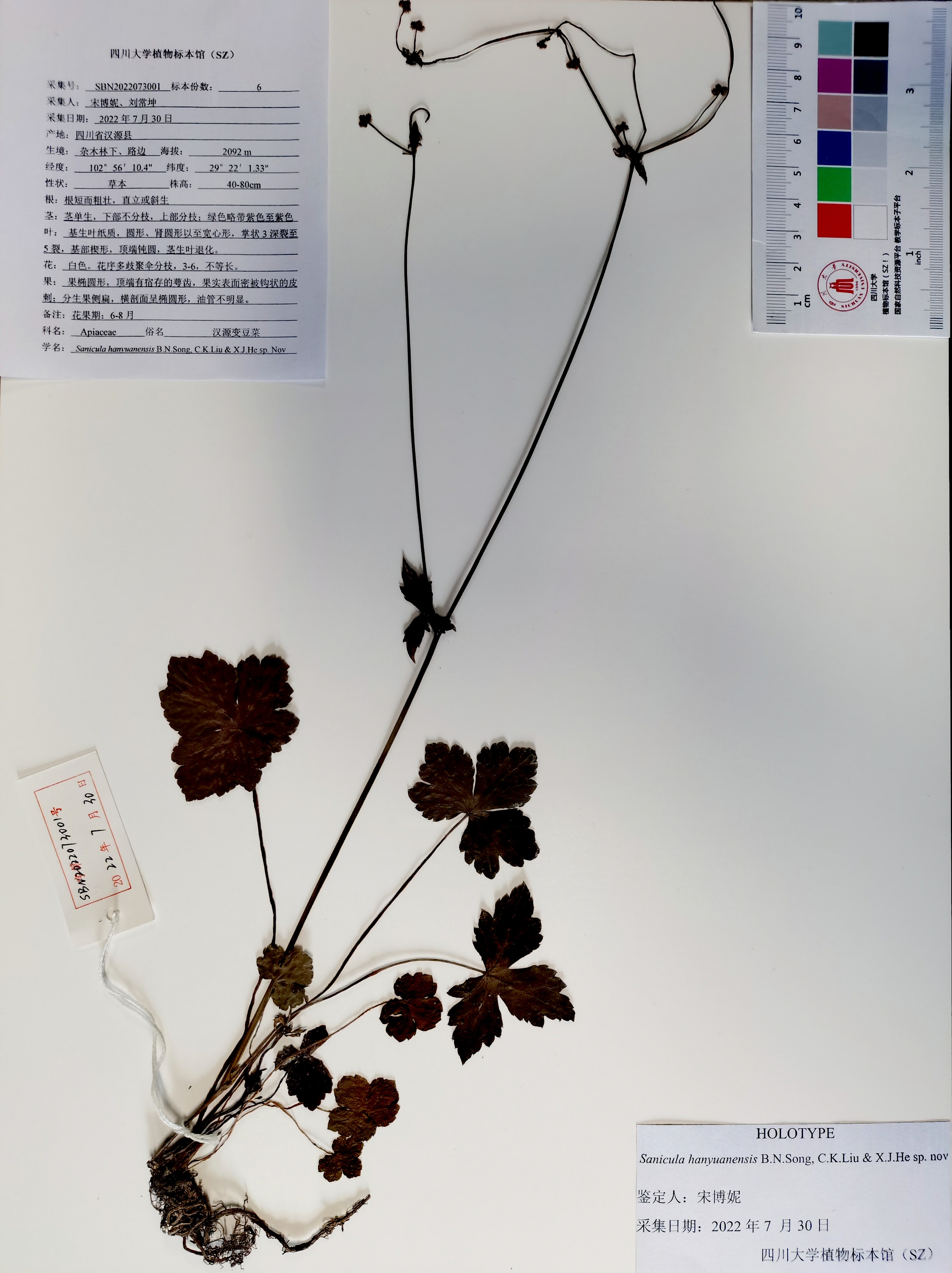

Supplement: Supplementary Figure 8 — Holotype of Sanicula sp. SBN2022073001. [file Image_8.jpeg]

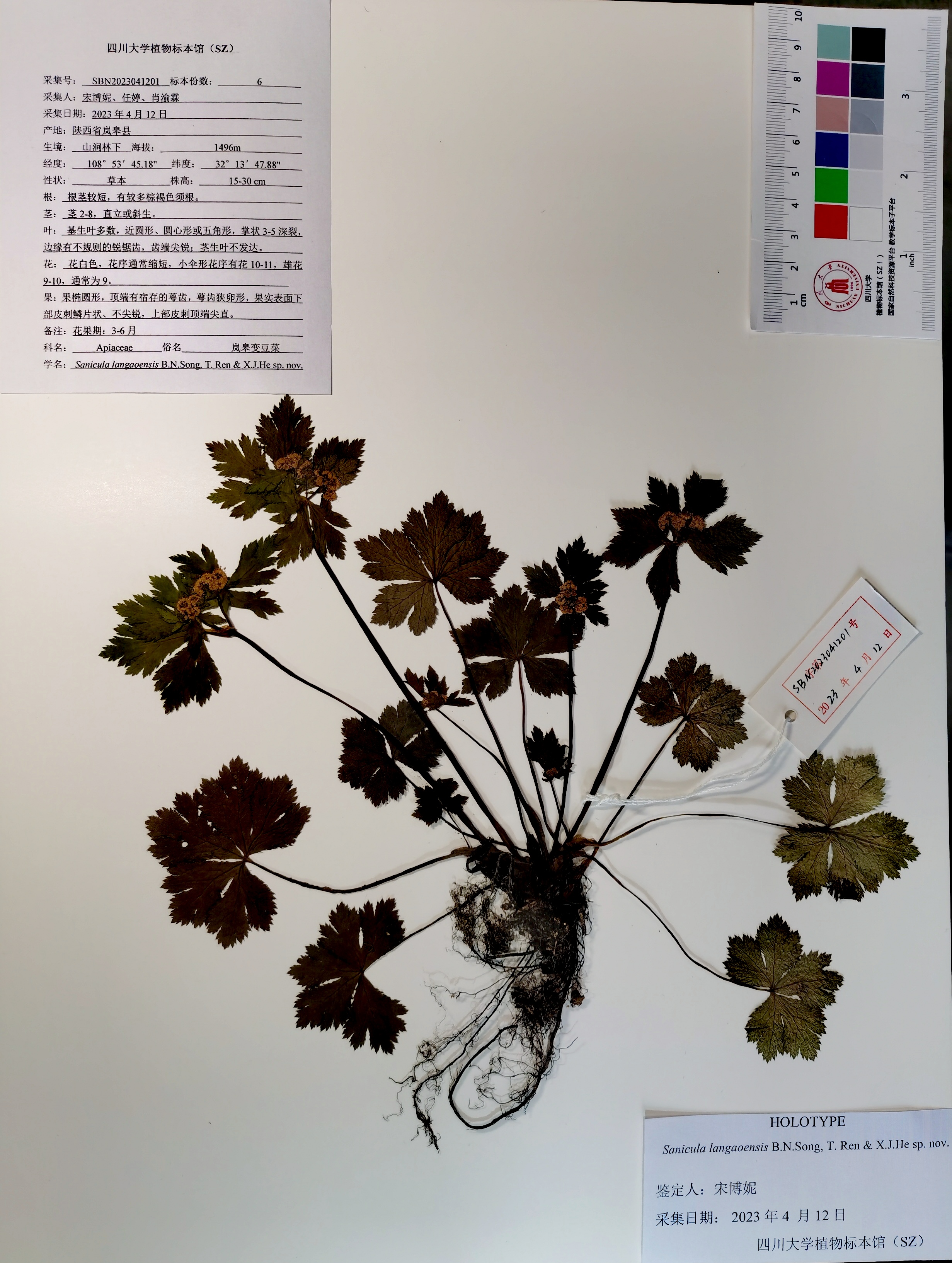

Supplement: Supplementary Figure 9 — Holotype of Sanicula sp. SBN2023041201. [file Image_9.jpeg]
