# Supplementary material for: Plastid phylogenomics contributes to the taxonomic revision of taxa within the genus Sanicula L. and acceptance of two new members of the genus
Source: Front Plant Sci. 2024 Jun 10;15:1351023. doi: 10.3389/fpls.2024.1351023 (PMC11194442; doi:10.3389/fpls.2024.1351023)
Supplement: Supplementary Table 3 — Comparison of plastome features among 21 Sanicula plants. [file Table_3.doc]

**Table S3. Comparison of plastome features among 21 *Sanicula* plants.**

| Taxon | Length (bp) | | | | GC contents (%) | | | | Number of genes (unique) | | | |
| --- | --- | --- | --- | --- | --- | --- | --- | --- | --- | --- | --- | --- |
|  | Genome | LSC | SSC | IR | Total | LSC | SSC | IR | Total | PCGs | rRNA | tRNA |
| *S. astrantiifolia* | 155,748 | 86,140 | 17,096 | 26,256 | 38.10 | 36.4 | 32.5 | 42.9 | 113 | 79 | 4 | 30 |
| *S. chinensis* | 155,395 | 85,677 | 17,072 | 26,322 | 38.20 | 36.5 | 32.6 | 42.9 | 113 | 79 | 4 | 30 |
| *S. caerulescens* | 155,768 | 86,174 | 17,118 | 26,238 | 38.20 | 36.4 | 32.6 | 42.9 | 113 | 79 | 4 | 30 |
| *S. elongata* | 155,634 | 86,051 | 17,105 | 26,239 | 38.20 | 36.4 | 32.5 | 42.9 | 113 | 79 | 4 | 30 |
| *S. flavovirens* | 155,335 | 85,682 | 17,049 | 26,217 | 38.20 | 36.4 | 32.5 | 43.0 | 113 | 79 | 4 | 30 |
| *S. hacquetioides* | 155,494 | 85,771 | 17,075 | 26,324 | 38.20 | 36.5 | 32.5 | 42.9 | 113 | 79 | 4 | 30 |
| *S. lamelligera* | 155,708 | 86,122 | 17,106 | 26,240 | 38.20 | 36.4 | 32.5 | 42.9 | 113 | 79 | 4 | 30 |
| *S. oviformis* | 155,645 | 86,071 | 17,098 | 26,238 | 38.20 | 36.4 | 32.6 | 42.9 | 113 | 79 | 4 | 30 |
| *S. pengshuiensis* | 155,765 | 86,189 | 17,102 | 26,237 | 38.20 | 36.4 | 32.6 | 42.9 | 113 | 79 | 4 | 30 |
| *S. rubriflora* | 155,657 | 85,982 | 17,057 | 26,308 | 38.20 | 36.4 | 32.5 | 42.9 | 113 | 79 | 4 | 30 |
| *S. rugulosa* | 155,527 | 85,780 | 17,079 | 26,334 | 38.20 | 36.5 | 32.5 | 42.9 | 113 | 79 | 4 | 30 |
| *S. serrata* | 155,599 | 86,046 | 17,097 | 26,228 | 38.20 | 36.5 | 32.4 | 42.9 | 113 | 79 | 4 | 30 |
| *S. odorata* | 154,500 | 85,074 | 17,074 | 26,176 | 38.20 | 36.5 | 32.5 | 42.9 | 113 | 79 | 4 | 30 |
| *S. giraldii* | 155,609 | 85,857 | 17,086 | 26,333 | 38.20 | 36.5 | 32.5 | 42.9 | 113 | 79 | 4 | 30 |
| [*S. giraldii* var*. ovicalycina*](http://www.iplant.cn/info/Sanicula giraldii var. ovicalycina?t=z) | 155,792 | 86,218 | 17,094 | 26,240 | 38.20 | 36.4 | 32.6 | 42.9 | 113 | 79 | 4 | 30 |
| *S. tienmuensis* | 155,717 | 86,075 | 17,106 | 26,268 | 38.20 | 36.4 | 32.5 | 42.9 | 113 | 79 | 4 | 30 |
| [*S. tienmuensis* var*. pauciflora*](http://www.iplant.cn/info/Sanicula tienmuensis var. pauciflora?t=z) | 155,693 | 86,145 | 17,066 | 26,241 | 38.20 | 36.4 | 32.5 | 42.9 | 113 | 79 | 4 | 30 |
| *S. orthacantha* | 155,749 | 86,166 | 17,103 | 26,240 | 38.20 | 36.4 | 32.6 | 42.9 | 113 | 79 | 4 | 30 |
| *S. orthacantha* var*. stolonifera* | 155,396 | 85,818 | 17,098 | 26,240 | 38.20 | 36.4 | 32.5 | 42.9 | 113 | 79 | 4 | 30 |
| *Sanicula sp*. SBN2022073001 | 155,676 | 86,012 | 17,104 | 26,280 | 38.20 | 36.4 | 32.5 | 42.9 | 113 | 79 | 4 | 30 |
| *Sanicula sp.* SBN2023041201 | 155,739 | 86,133 | 17,104 | 26,251 | 38.20 | 36.4 | 32.5 | 42.9 | 113 | 79 | 4 | 30 |
